# Genes Contributing to Resilience and Sensitivity to Lisinopril at Old Age: Clinical Translation of GWA in Drosophila

**DOI:** 10.1093/geroni/igab046.2555

**Published:** 2021-12-17

**Authors:** Mariann M Gabrawy, Nick Khosravian, George S Morcos, Meagan Jezek, Jeremy Walston, Peter M Abadir, Jeff Leips

**Affiliations:** 1 Johns Hopkins University School of Medicine, Baltimore, Maryland, United States; 2 University of Maryland Baltimore County, Baltimore, Maryland, United States; 3 University of Maryland School of Medicine, Baltimore, Maryland, United States

## Abstract

Despite impressive results in restoring physical performance in rodent models, treatment with Renin-Angiotensin System (RAS) inhibitors such as Lisinopril have highly mixed results in humans, likely, in part, due to genetic variation in human populations. To date, the genetic determinants of responses to drugs such as RAS inhibitors remain unknown. Given the complexity of the relationship between physical traits and genetic background, genomic studies which predict genotype- and age-specific responses to drug treatments in humans or vertebrate animals are difficult. Here, using 126 genetically distinct lines of Drosophila, we tested the effects of Lisinopril on climbing speed and endurance at young and old age (N=14,310). Our data show that functional response and sensitivity to Lisinopril ranges from significant protection against physical decline (8–100% faster, P< 0.0001) to increased weakness (P< 0.0001) depending on both genotype and age (P< 0.0001). Genome-wide analyses revealed little to no overlap in candidate polymorphisms influencing sensitivity between ages nor between treatments within each age. Furthermore, network analyses led to identification of evolutionarily conserved genes in the WNT signaling pathway as being significantly associated with variations in sensitivity to Lisinopril. Genetic knockdown of Axin, frizzled, nemo, and wingless, genes with human orthologs AXIN1, FZD1, NLK, and WNT1, respectively, abolished the effects of Lisinopril treatment. Our results implicate these genes as contributors to the genotype- and age-specific effects of Lisinopril treatment and as potential therapeutic targets for improvement of resiliency. Our approach should be widely applicable for identifying genomic variants that predict age-dependent responses to pharmaceutical treatments.

